# Lung Ultrasonography Is an Acceptable Imaging Modality to Diagnose COVID-19 and Effectively Correlates with HRCT Chest—A Prospective Study

**DOI:** 10.3390/diagnostics13122091

**Published:** 2023-06-16

**Authors:** Muiez Bashir, Wani Inzamam, Mohd Kamran Banday, Sheikh Riaz Rasool, Mudasir Hamid Bhat, Carmen Vladulescu, Fahad A. Al-Misned, Hamed A. El-Serehy

**Affiliations:** 1 Department of Radiology, Sher-e-Kashmir Institute of Medical Sciences Soura, Jammu & Kashmir, Srinagar 190011, India; 2 Department of General Surgery, Sher-e-Kashmir Institute of Medical Sciences Soura, Jammu & Kashmir, Srinagar 190011, India; 3 Department of Biology and Environmental Engineering, University of Craiova, 200585 Craiova, Romania; 4 Department of Zoology, College of Science, King Saud University, Riyadh 11451, Saudi Arabia

**Keywords:** lung ultrasonography, RT–PCR, COVID-19, disease, diagnosis, HRCT

## Abstract

It has been validated beyond doubt that High-Resolution Computed Tomography (HRCT) chest and to some extent chest radiographs have a role in corona virus disease-19 (COVID-19). Much less is known about the role of lung ultrasonography (LUS) in COVID-19. In this paper, our main purpose was to gauge the relationship between LUS and chest HRCT in reverse transcriptase polymerase chain reaction (RT–PCR) documented cases of COVID-19, as well as in those with high suspicion of COVID-19 with negative RT–PCR. It was a prospective study carried out at our tertiary care hospital, namely, SKIMS Soura. The total number of patients in this study were 152 (200 patients were selected out of which only 152 had undergone both LUS and chest HRCT). The patients were subjected to both LUS and chest HRCT. The radiologist who performed LUS was blinded to clinical findings and HRCT was evaluated by a radiologist with about a decade of experience. The LUS findings compatible with the disease were subpleural consolidations, B-lines and irregular pleural lines. Findings that were compatible with COVID-19 on chest HRCT were bibasilar, subpleural predominant ground glass opacities, crazy paving and consolidations. COVID-19-positive patients were taken up for chest HRCT for disease severity stratification and were also subjected to LUS. On HRCT chest, the imaging abnormalities compatible with COVID-19 were evident in 110 individuals (72.37%), and on Lung Ultrasound they were observed in 120 individuals (78.95%). Imaging of COVID-19 patients assessed by both LUS and HRCT chest,, showed a positive correlation (*p* < 0.0001). The study revealed a sensitivity of 88%, a specificity of 76.62%, a positive predictive value of 78.57% and a negative predictive value of 86.76%. None of the individuals with a diagnosis of COVID-19 on HRCT were missed on LUS. An excellent correlation was derived between the LUS score and CT total severity score (*p* < 0.0001 with a kappa of 0.431). Similar precision compared with chest HRCT in the detection of chest flaws in COVID-19 patients was obtained on LUS.

## 1. Introduction

The severe acute respiratory syndrome coronavirus 2 (SARS-CoV-2) is the agent that causes coronavirus disease 2019 (COVID-19). The illness began in Wuhan, China in December 2019 and quickly spread to the rest of the world. Being highly infectious, it spread to more than 180 nations, prompting the World Health Organization to proclaim it as a pandemic on 11 March 2020 [1]. Until now 662,445, 150 confirmed cases and 6,704,827 fatalities have occurred since the onset of the pandemic. The need of the hour was fast confirmation and characterization of suspected cases since emergency departments (EDs) were already dealing with an enormous inflow of patients and scarce medical facilities. Reverse Transcriptase Polymerase Chain Reaction (RT–PCR) of the SARS-CoV-2 nucleic acid is the primary diagnostic approach; nevertheless, it has several drawbacks, including limited sensitivity and technical challenges in the test’s execution [2]. Chest radiography was used early at the start of the disease. The most characteristic radiographic appearance, despite its limited sensitivity (missing in more than 40% of patients), is the presence of patchy/diffuse infiltrates on the chest radiograph. Peripheral ground glass opacities affecting the lower lobes is the most frequent finding on chest x-rays for COVID-19 patients, accounting for nearly half of all aberrant findings. Regarding the use of radiography in the evaluation of COVID-19 patients, little is known. Radiographs were examined to determine the presence of pleural effusion, reticulations, nodular consolidations and ground glass opacities.

The radiographic assessment of lung edema (RALE) score, developed by Warren et al., was used to determine the severity score. Ground glass consolidation or opacity was graded on a scale of 0 to 4 (0 = no involvement, 1 = less than 25%, 2 = 25–50%, 3 = 50–75%, and 4 = more than 75%). Total score was calculated as the sum of all scores. The most frequent radiographic findings were consolidations and ground glass opacities [3]. Since the pandemic began, it has been proven beyond doubt that High-Resolution Computed Tomography (HRCT) scan is the primary imaging test because of its increased sensitivity to depict ground glass opacities (GGO’s) [4]. Patients with COVID-19 frequently have bilateral lung field development, multilobar and peripheral lung involvement, and a prevalence of posterior lung involvement. Ground-glass opacities, crazy paving, interlobular septal thickening, bronchiectasis and halo sign are examples of frequently seen radiological findings. Less often occurring ones include mediastinal adenopathy, pleural effusion and pericardial effusion. Even vascular tissues were not immune to this sickness [5]. Radiation exposure, excessive use of medical resources, or difficulties in obtaining a CT scan appear to outweigh the necessity for treatment for minor illnesses [6]. Radiation safety continues to be the top concern for everyone in the medical field, including patients, physicians and other staff members. Radiology, radiation oncology, interventional cardiology and surgical professionals are among the departments at risk. The biggest radiation dose currently received by medical professionals comes through fluoroscopy, with diagnostic radiology making up the smallest portion of the total radiation exposure. Any radiation dose, whether it comes from a patient or a worker, is dangerous [7]. Although the level of monitoring for each patient admitted to the ICU ought to be consistent, it might vary. Perhaps all that is needed for hemodynamically stable patients is continuous electrocardiographic (ECG) monitoring, routine non-invasive blood pressure checks, and peripheral pulse oximetry (peripheral oxygen saturation or SpO2). For continual invasive blood pressure monitoring and routine study of arterial blood gases in patients who are unstable or at risk of becoming unstable, an arterial line should be inserted. Additionally, in critically sick patients, it may not be possible to subject them to chest HRCT as their hemodynamic instability may preclude it. Thus, alternative imaging modalities which can be performed within critical care settings will be highly useful [8]. Ultrasonography (USG) machines, which are widely available, can be used in such settings to carry out LUS. Moreover, LUS can be accomplished very quickly [9]. We are well aware of the usefulness of LUS to diagnose lung pathologies, however, its usefulness in COVID-19 patients is very limited and only a few studies and case reports are available as of now [10]. The LUS findings which are highly indicative of COVID-19 are the presence of subpleural consolidations, an irregular pleural line and B-lines [11]. There is no doubt about the usefulness of chest HRCT in COVID-19 patients, however, the role of LUS in COVID-19 has not been fully validated [12].

## 2. Materials and Methods

### 2.1. Initial Patient Assessment

The selected patients were evaluated and data in the form of medical history (symptomatology, demographic data and presence or absence of comorbidities), physical exam (oxygen saturation and respiration rate), and laboratory evaluation (complete hemogram, baseline investigations, CRP, ferritin, INR were performed). Chest HRCT scans were acquired using a 64-slice multidetector CT (SOMATOM, Siemens Health liners). Scans were obtained with patients in a supine position and at the end inspiration. The following parameters were used to acquire and reconstruct image data: 1 mm section thickness, 0.6-mm recon interval, 120 kVp. In younger patients and lean adult ones, a low-dose CT protocol was used (100 kVp). The review was carried out by an experienced radiologist (with about a decade of experience) who was blinded to clinical findings. The lung lobes were scored as 0, 1, 2, 3 and 4 for disease involvement as nil (0%), minimal (1–25%), mild (26–50%), moderate (51–75%) and severe (76–100%), respectively. The sum of five lobe scores was summed to obtain a final CT Severity Score (CTSS) (range: 0–20).

**Figure 1:**
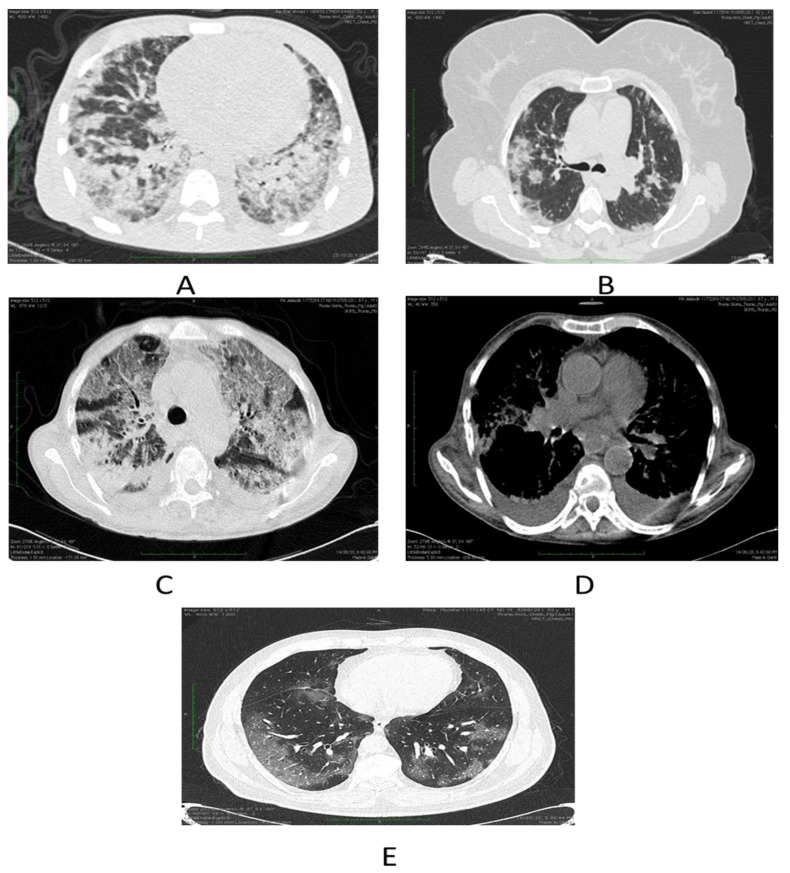
(**A**) Depicts severe COVID-19 pneumonia in a patient in form of consolidations. (**B**) Shows few patches of nodular consolidations in a patient with moderate disease. (**C**) Demonstrates combined ground glass opacities and consolidations in a patient with severe COVID-19 illness. (**D**) Axial chest image bespeaks bilateral effusions. (**E**) Ground glass opacities indicated by axial chest cuts.

Chest HRCT scans were assessed for the existence and dissemination of ground-glass opacities (GGO’s) which are taken as areas of haziness inadequate to obscure the underlying vascular markings, septal thickening (intra or interlobular), crazy paving, consolidations and other atypical findings favoring COVID-19. The presence of many lobar or patchy ground glass opacities, with or without thickening of the interlobular septae (crazy paving), consolidations, and preponderance of the subpleural and bibasilar tissues, is highly symptomatic of COVID-19 disease (Figure 1).

Three patterns of lung involvement are seen (i) Patchy or diffuse GGO’s; (ii) Inhomogeneously distributed collapse and peribronchial opacities; (iii) ARDS -like pattern. Chest radiographs, if available were also assessed and data was collected.

### 2.2. Ultrasound Data Collection

**Figure 2:**
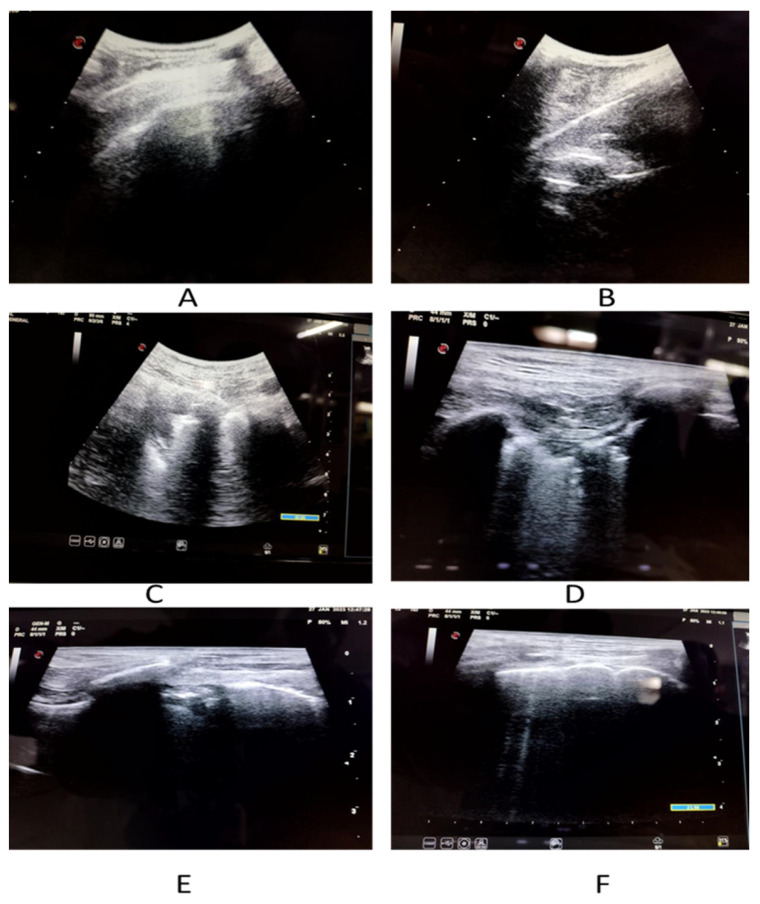
Imaging spectrum of COVID-19 patients on LUS. (**A**) Shows thickened pleura with small pleural effusion. (**B**) Depicts thickened irregular pleural line. (**C**) Demonstrates confluent B-lines. (**D**) Reveals isolated as well as confluent B-lines. (**E**) Displays thickened irregular pleural line with confluent B-lines. (**F**) Denotes isolated B-lines.

**Figure 3:**
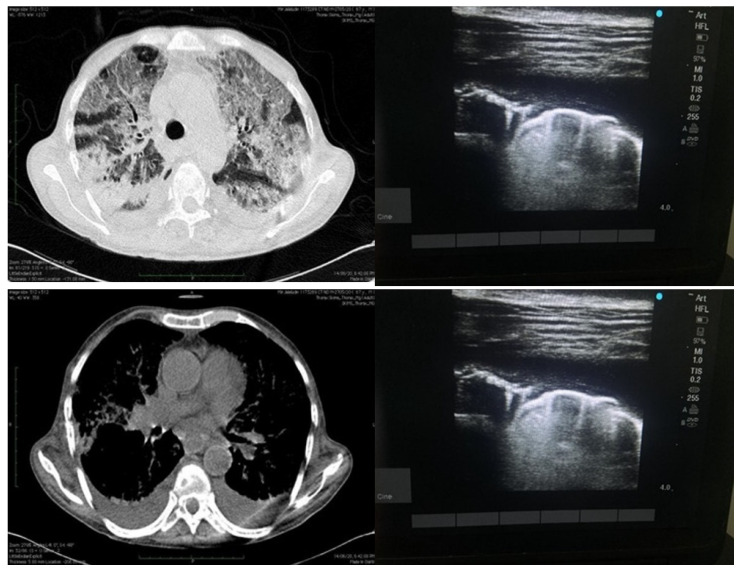
Depicting findings of LUS on right side (above and below) as effusion with irregular pleural line and isolated B-lines and finally small effusion with isolated B-lines as well as confluent B-lines. Image on the left side depicts same patient with HRCT findings of consolidations as well ground glass opacities and pleural effusion.

An experienced sonologist who had knowledge of performing and elucidating LUS performed all the examinations. The LUS exam was carried out in accordance with a 12-zone protocol. Careful evaluation was done to look for pleural effusions, confluent B-lines (CBL), isolated B-lines (IBL), irregular pleural line (IPL) and consolidations (Figure 2 and Figure 3). Both halves of the chest were scanned on their anterior, lateral and posterior aspects. These 12 zones were evaluated for the isolated B-lines (1 point), confluent B-lines (2 points) and consolidations or pleural effusion (3 points) with score range from (0–3). By summing the scores of all 12 zones the total lung score was achieved (range of possible scores: 0–36).

Pattern of B-lines, either solitary and/or confluent, an uneven pleural line, and subpleural consolidations are all regarded as imaging signs of a compatible LUS exam. LUS examinations were performed using a GE Logic p5 USG machine, using a curvilinear array transducer and lung/cardiac pre-set. The sonologist performing the scan was blinded to all clinical details and HRCT findings.

### 2.3. Outcome Measures and Definitions

The primary goal of our study was to determine whether there was a relationship between sonographic evaluation of lung and high-resolution chest CT in COVID-19 patients and how well it could diagnose illness in these patients. A positive RT–PCR test and clinically dubious but negative RT–PCR were considered as cases of COVID-19.

### 2.4. Statistical Analysis

The presentation of the categorical variables was performed in the form of number and percentage (%). On the other hand, the quantitative data with normal distribution were presented as the means ± SD and the data with non-normal distribution as median with 25th and 75th percentiles (interquartile range). The data normality was checked by using the Kolmogorov–Smirnov test. In the cases in which the data were not normal, we used non parametric tests. The following statistical tests were applied for the results: The comparison of the variables which were quantitative and not normally distributed in nature were analyzed using the Kruskal–Wallis test and variables which were quantitative and normally distributed in nature were analyzed using ANOVA. The comparison of the variables which were qualitative in nature were analyzed using Fisher’s exact test as at least one cell had an expected value of less than 5. Inter-rater kappa agreement was used to assess strength of agreement between lung ultrasonography and computed tomography. Sensitivity, specificity, positive predictive value and negative predictive value of lung ultrasonography was calculated for predicting severity after taking computed tomography as the gold standard. The data entry was done in a Microsoft EXCEL spreadsheet and the final analysis was done with the use of Statistical Package for Social Sciences (SPSS) software, IBM manufacturer, Chicago, IL, USA, ver 25.0. For statistical significance, *p* value of less than 0.05 was considered statistically significant.

### 2.5. Aim

The primary objective of this study was to define the role of LUS in COVID-19 patients. This was a prospective study which was carried out in SKIMS, Soura. IEC committee approval was obtained. Each enrolled patient was informed prior to inclusion in the study and proper consent was taken. All those patients who had positive RT–PCR and those with clinically high suspicion of COVID-19 illness, and in whom RT–PCR couldn’t detect illness, and were subjected to chest HRCT for evaluation, were included in the study. The main indication for subjecting patients to chest HRCT was a high chance of COVID-19 illness and negative RT–PCR. We excluded patients who refused to undergo chest HRCT and who were <18 yrs.

## 3. Results

In our study, we had 152 patients (200 patients selected, however only 152 patients had both LUS and HRCT scans). The study was carried out from March to July 2021 (Table 1).

Among these patients admitted to the ICU, six passed away (3.9%). Approximately more than half of these patients (84 patients, 55.2%) had a chest radiograph, of which 43% were negative (Table 2). The most common imaging sign was (ground glass opacities) GGO’s as were seen in 110 patients (72.3%), followed by thickened inter and intralobular septae in 53 patients (34.9%), (Table 2). Only central involvement of lung parenchyma was seen in six patients (3.95%), amongst which four patients had cardiac failure and two patients had viral/infectious bronchiolitis.

Subpleural consolidations involving the posterior lower lobes was the common imaging finding depicted by LUS (Table 2). Mean lung ultrasonography (LUS) score in our study was 11.60 (SD = 4.3) and computed tomographic severity score (CTSS) was 11.63 (SD = 4.06). When compared to CTSS, the LUS score corresponded well, (CI = 95%, *p* < 0.0001). The severity of the lesions demonstrated by LUS and CTSS are described in (Table 3).

Age, LUS and CTSS did not have any statistical significance (*p*-value 0.468). On computed tomographic (CT) scans, 110 patients (or 72.3%) and on LUS 120 patients (or 78.9%) had radiologic indications that were indicative of, or very consistent with, COVID-19 (Table 2). Data analysis revealed that our study has a sensitivity of 88%, a specificity of 76.62%, a positive predictive value of 78.57%, and a negative predictive value of 86.76%, all patients with abnormal findings on CT were properly identified with LUS (CI > 95%, *p* < 0.0001). The diagnostic accuracy was 82.24% (Table 4).

To test if chest X-ray, LUS and chest CT scans detected COVID-19 aberrant lung abnormalities, Cohen’s k was used. Chest CT and LUS showed excellent agreement (k = 0.43, *p* < 0.0001). Chest radiography findings and CT scan results did not statistically substantially agree. Chest X-ray findings were only sporadically linked with LUS results. Only two patients exhibited phenotype 3 (1.82%), the majority of patients had chest CT phenotypes 1 (*n* = 52, 47.27%) and 2 (*n* = 25, 22.73%). Despite the fact that 120 patients showed LUS results that were consistent with COVID-19, 6 patients had infectious bronchiolitis, and none had metastatic lung illness. As a result, in our sample, there was no error in diagnosing COVID-19 using LUS as opposed to CT.

## 4. Discussion

We discovered a strong relationship linking High Resolution Computed Tomography (HRCT) chest and Lung Ultrasonography (LUS). None of the positive computed tomographic manifestations were depicted as negative on lung ultrasonography. This method has an extremely low quantity of false-negative rates, which is crucial in the era of the current pandemic. There is a growing body of research on the difficulties in diagnosing COVID-19 illness (Shi et al., 2020; Zhang et al., 2020) [13]. Imaging modalities play a key role in the oversight of these patients, in whom RT–PCR is negative despite having high symptomatology favoring COVID-19. By identifying the early stages of respiratory infections, CT scanning allows for a prompt public safety response. Chest HRCT has been found to be the primary source of information for acceptable findings. We are well aware of this because chest radiography is ineffective at detecting ground glass opacities, it is not the preferred imaging technique for diagnosing COVID-19 disease. Additionally, chest radiography is generally normal, particularly in the early stages of sickness [14]. An analysis of 1049 patients who underwent a chest HRCT scan and a RT–PCR test revealed that CT aberrations had a high reliability for diagnosing COVID-19 patients, indicating that an HRCT scan should be used as a screening tool [15]. The use of CT scans in emergency departments, however, is constrained by a number of factors, including radiation dose, which is particularly problematic for minor illnesses, a lack of its availability, and a prohibition on using them on unstable patients [16]. As has been proven in various studies (30.8%), our study also had good number of patients with normal chest HRCT (27.6%) (Li et al., 2020) [17]. According to early publications during COVID-19, LUS imaging signs agree well with chest HRCT scan findings. LUS can safely rule out clinically significant COVID-19 infection and may help with COVID-19 diagnosis in environments with a high frequency of cases [18]. Additionally, Soldati et al. suggested a systematized method for carrying out LUS in these patients, which included a 14-zone methodology and scoring to gauge the degree of chest involvement. The lung ultrasonography score is significantly greater in community-acquired pneumonia than it is in SARS-CoV-2 disease [19]. Although we concur that there should be agreement on the LUS exam procedure, the 12-zone strategy has received greater attention and has been shown to be effective (Cantinotti et al., 2020) [20]. According to studies, individual rates of heart injury from COVID-19 range from 7.2% to 14%. In a study of 121 individuals who had cardiovascular problems from SARS, 61 individuals (50.4%) in the institution had hypertension. Of these individuals, 71.9% experienced chronic tachycardia, with 40% experiencing it even after being discharged from the hospital. Despite the fact that the risk of cardiovascular issues like tachycardia is substantially higher when SARS-CoV-2 is present, these issues were clinically inconsequential and unconnected to the increased mortality risk. In this investigation, more individuals with cardiac injury died while still in the hospital than did so with SARS, proving that COVID-19-induced cardiac injury is linked to very negative clinical outcomes. The exact cause of cardiac damage in these COVID-19 patients is currently unknown, however, the human cell receptor angiotensin-converting enzyme 2 (ACE2), which is also abundantly expressed in the heart, has a great affinity for the spike protein of SARS-CoV-2. It makes sense to speculate that ACE2 may act as a mediator of the heart damage caused by COVID-19 [21].

In our data six patients showed conditions different than the conventional COVID-19 (central distribution) findings: two patients experienced viral bronchiolitis, and four patients had cardiac involvement. These patients had some peripheral lung involvement, which was observed on LUS and was labelled as suggestive of COVID-19, despite having mostly central lung involvement on CT. Low specificity is one of the primary drawbacks of LUS, since the results may overlap with those of other respiratory disease etiologies, such as viral diseases, bacterial pneumonias, lung infarction, and distant metastatic disease. A similar restriction could be applicable to CT scans, which might mistake COVID-19 for other viral pneumonias. Nevertheless, in this pandemic, positive LUS or HRCT characteristics might still be strongly predictive of COVID-19 infection even in the absence of a negative RT–PCR result. Handheld US devices which are user friendly, economical, easily available and far easier to disinfect can be used to gauge severity of lung involvement and its follow up. There are many advantages in conducting LUS over HRCT scans, in particular for population subgroups, such as expecting females and young. Additionally, it prevents the patient with possible COVID-19 from being sent to imaging and thus threatens the life of other individuals or healthcare dispensers). In our research, we found a significant association between LUS and chest HRCT aberrations that are indicative of lung parenchymal affliction brought on by SARS-CoV-2 infection. According to a prior publications, the majority of patients (92.5%, 37 of 40 patients with aberrant CT results) had peripheral involvement of both lungs, which is detectable with ultrasonography. Notably, the LUS score had a stronger correlation with age, physical evaluation (oxygen levels), and other well-established inflammatory markers (like CRP) that have been shown to be more useful in this situation than the CTSS. Although it may imply that a 12-zone LUS score, has higher portrayal of posterior lung fields in half of the lung zones (in comparison to a 5-lobe division, with the depiction of posterior lung fields in two zones only), and it better reflects the patient’s physiologic state, this higher correlation should be interpreted with caution. Chest CT characteristics vary between individuals, allowing for the establishment of unique phenotypes that may direct treatment and ventilator settings. The type 1 phenotype patient may gain from the utilization of positive end-expiratory pressure (PEEP) and is expected to respond well to inhaled Nitric Oxide. In the type 2 phenotype, prone posture and moderate to high PEEP may aid in recruiting collapsed zones. The management of the type 3 phenotype should be similar to that of standard ARDS. Consequently, LUS can be considered an established modality of imaging to diagnose COVID-19 and may also prove to be a reliable means of diagnosing disease in future waves, if they occur. We assessed the radiological load (CT and LUS) in light of the clinical signs, laboratory findings, and outcomes.

### Limitations

The primary drawback is that LUS generally lacks specificity. LUS imaging signs of COVID-19 imbricate when compared to other pneumonia etiologies or non-pneumonia illnesses (e.g., interstitial lung disease). However, in places where the COVID-19 virus is widespread, positive LUS characteristics can still be very indicative of infection even in the absence of positive RT–PCR or chest radiograph results. As a consequence, as the frequency and incidence of COVID-19 infection declines, the findings of this study offer a chance to further examine the effectiveness of ultrasound in various contexts and clinical scenarios. The outcome of our study gives a chance to further study LUS in various clinical contexts especially as COVID illness has significantly eased off. Any research work has some amount of selection bias and our study was no exception. The limited generalizability of our findings is due to the fact that the expert sonographer only performed all ultrasounds during his working hours. Another drawback of LUS or chest HRCT findings might have been evaluated in the early stages of the illness, much prior to parenchymal affliction, as a result, diagnostic modalities must be taken as an adjunct to RT–PCR and other laboratory studies in any patient. The LUS or CT results were not correlated with patient outcomes. We see LUS as a feasible remedy and advise that it can be used as the initial imaging tool and for a subsequent look at COVID-19 patients, especially in a low-resource country like ours.

## 5. Conclusions

LUS is an accurate imaging modality which helps in identifying lung abnormalities in COVID-19 patients, as is HRCT chest. This further escalates given the fact that the COVID-19 pandemic overpowered the most developed countries and severely dented the healthcare options in low-resource countries. Thus, utilizing LUS could be a solution in future waves, if and when they occur.

## Figures and Tables

**Table 1:** Distribution of demographic and baseline characteristics of study.

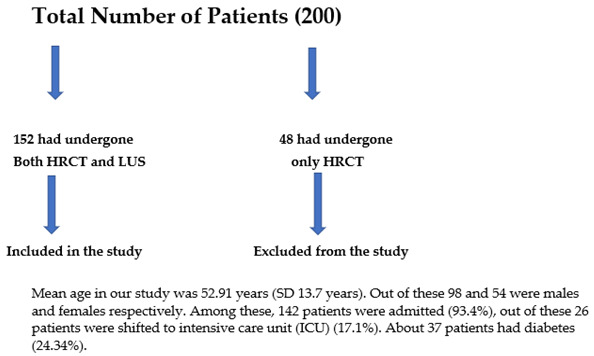		
Demographic and Baseline Characteristics		
Age (years)	Frequency	Percentage
<=20 years	01	0.66%
21–40 years	26	17.11%
41–60 years	86	56.58%
61–80 years	37	24.34%
>80 years	02	01.32%
Mean ± SD	52.9 ± 13.7	
Median (25th–75th percentile)	50 (45–61.25)	
Range	18–88	
Gender		
Female	54	35.53%
Male	98	64.47%
Diabetics	37	24.34%

**Table 2:** Imaging modality distribution and affected zone distribution.

Imaging Modality			*n* (%)
Chest Computed Tomography (*n* = 152)			
COVID-19 suggestive			110 (72.37)
Pleural thickening			3 (1.97)
Ground-glass opacity			110 (72.37)
Septal thickening			53 (34.87)
Crazy paving			30 (19.74)
Subpleural consolidation			30 (19.74)
Pleural effusion			36 (23.68)
COVID-19 phenotypes (*n* = 110)			
Phenotype 1			52 (47.27)
Phenotype 2			25 (22.73)
Phenotype 3			2 (1.82)
Distribution (*n* = 152)			
Peripheral			69 (45.39)
Diffuse			21 (13.82)
Central and peripheral			21 (13.82)
Central			6 (3.95)
Normal			36 (23.68)
CT total severity score (Mean ± SD)			11.63 ± 4.06
Mild			77 (50.66)
Moderate			68 (44.74)
Severe			7 (4.61)
CT pulmonary angiogram (*n* = 152)			63 (41.45)
Pulmonary embolism			21 (13.82)
Lung ultrasonography (*n* = 152)			
COVID-19 suggestive			120 (78.95)
Right pleural effusion			24 (15.79)
Left pleural effusion			21 (13.82)
Pericardial effusion			39 (25.66)
Lung score, (Mean ± SD)			11.6 ± 4.3
Chest X-ray results, *n* = 84			
COVID-19 suggestive			48 (57.14)
Ground-glass opacity			36 (42.86)
Interstitial pattern			39 (46.43)
Affected Zone	IP/IBL	CBL	C
1 (right upper anterior)	27	12	24
2 (right lower anterior)	36	27	9
3 (right upper lateral)	30	30	12
4 (right lower lateral)	42	30	9
5 (left upper anterior)	33	12	18
6 (left lower anterior)	27	12	15
7 (left upper lateral)	24	24	18
8 (left lower lateral)	39	27	6
9 (right upper posterior)	24	18	27
10 (right lower posterior)	39	15	57
11 (left upper posterior)	21	15	18
12 (left lower posterior)	39	15	54

**Table 3:** Inter-kappa agreement between LUS and CTSS.

LUS	CTSS			Total	Kappa	*p*-Value
	Mild 0–11	Moderate 12–18	Severe >18			
Mild	59	07	02	68		
Moderate	12	37	02	51	0.431	<0.0001
Severe	06	24	03	33		
Total	77	68	07	152		

**Table 4:** Depicting Sensitivity, Specificity, PPV, NPV, AUC, and Diagnostic accuracy.

Computed Tomography	Lung Ultrasonography
Sensitivity (95% CI)	88% (78.44% to 94.36%)
Specificity (95% CI)	76.62% (65.59% to 85.52%)
AUC (95% CI)	0.82 (0.75 to 0.88)
Positive Predictive Value (95% CI)	78.57% (68.26% to 86.78%)
Negative Predictive Value (95% CI)	86.76% (76.36% to 93.77%)
Diagnostic accuracy	82.24%

## Data Availability

The data presented in this study are available on request from the corresponding author. The data are not publicly available due to privacy.
